# The role of neuropeptides in cutaneous wound healing: a focus on mechanisms and neuropeptide-derived treatments

**DOI:** 10.3389/fbioe.2024.1494865

**Published:** 2024-10-30

**Authors:** Liwei Xing, Bing Chen, Yuliang Qin, Xinyao Li, Sitong Zhou, Kai Yuan, Rong Zhao, Dongdong Qin

**Affiliations:** ^1^ First Clinical Medical College, Yunnan University of Chinese Medicine, Kunming, Yunnan, China; ^2^ School of Medicine, Kunming University, Kunming, China; ^3^ Key Laboratory of Traditional Chinese Medicine for Prevention and Treatment of Neuropsychiatric Diseases, Yunnan University of Chinese Medicine, Kunming, China; ^4^ Second Clinical Medical College, Yunnan University of Chinese Medicine, Kunming, Yunnan, China

**Keywords:** wound healing, neuropeptides, mechanisms, treatments, skin

## Abstract

An extensive network of cutaneous nerves, neuropeptides, and specific receptors richly innervates the skin and influences a variety of physiological and pathological processes. The sensory and autonomic nerve fibers secrete a variety of neuropeptides that are essential to the different phases of wound healing. In addition to initiating a neurogenic inflammatory response in the early stages of healing, neuropeptides also control wound healing by influencing immune cells, repair cells, and the growth factor network. However, the precise mechanism by which they accomplish these roles in the context of cutaneous wound healing is still unknown. Investigating the mechanisms of action of neuropeptides in wound healing and potential therapeutic applications is therefore urgently necessary. The present review discusses the process of wound healing, types of neuropeptides, potential mechanisms underlying the role of neuropeptides in cutaneous wound healing, as well as some neuropeptide-derived treatment strategies, such as hydrogels, new dressings, electro stimulation, and skin-derived precursors. Future in-depth mechanistic studies of neuropeptides in cutaneous wound healing may provide opportunities to develop therapeutic technologies that harness the roles of neuropeptides in the wound healing process.

## 1 Introduction

A wound is described as damage or disruption to normal anatomical structure and function. Wounds can cause harm to the tendons, muscles, arteries, nerves, bone, or even the skin’s epithelial integrity (Wilkinson and Hardman, 2020). According to extant studies, 15% of wounds are still unresolved one year after presentation, meaning that 3,500 people out of a population of 1 million would be suffering from wounds (Lindholm and Searle, 2016). The ‘silent epidemic’ of wounds is thought to account for 3% of total healthcare spending (Lindholm and Searle, 2016). A complex network of mediators, including neuropeptides, growth factors, and cytokines, participates in cutaneous wound healing. Wound healing is a dynamic process that involves inflammation, cell proliferation, and tissue remodeling (Wang et al., 2018). Neuropeptides are a family of extracellular messengers family that play a crucial role in wound healing by acting as neurotransmitters, hormones, or paracrine agents (Ashrafi et al., 2016). Numerous neuropeptides released by sensory and autonomic nerve fibers, such as Substance P (SP), calcitonin gene-related peptide (CGRP), neuropeptide Y (NPY), and vasoactive intestinal peptide (VIP) play a crucial role during the various phases of wound healing (Kiya and Kubo, 2019). This review discusses the process of wound healing, types of neuropeptides involved, potential mechanisms underlying the role of neuropeptides in wound healing, as well as some neuropeptide-derived treatment strategies aimed at promoting wound healing.

## 2 The process of wound healing

Although healing is a continuous process, it is divided into several phases to better understand the physiological processes occurring in the wound and its surrounding tissue.

### 2.1 Inflammation

A fibrin plug, which persists for a few hours, is created by cutaneous hemostasis. Neutrophils, which are the primary cellular components in the activation of the inflammatory phase of wound healing, are recruited by pro-inflammatory mediators released by aggregated platelets via chemotactic signals (Evers et al., 2010). Macrophages promote the persistence of inflammation by phagocytosing pathogens and cell debris as well as by secreting growth factors, chemokines, and cytokines, and by initiating the creation of granulation tissue (Wilkinson and Hardman, 2020). The inflammatory phase can take hours or days to subside.

### 2.2 Proliferation

Re-epithelialization, granulation tissue formation, and neovascularization are the hallmarks of the proliferative phase, which lasts many weeks (Pilcher et al., 1997). The extracellular matrix (ECM), which is made of type-3 collagen and produced by fibroblasts when macrophages secrete TGF-1 and PDGF, provides a structural foundation for endothelial cells, angiogenesis, and wound contraction (Profyris et al., 2012). Although the strong contractile force of myofibroblasts is necessary for physiological wound healing, it can be detrimental to tissue function if it causes an excessive buildup of ECM, which results in hypertrophic scarring (Hinz, 2007).

### 2.3 Remodeling

Scar maturation—which involves proliferative cell apoptosis, ECM disintegration and reconfiguration, and type-1 collagen replacement—distinguishes remodeling from the other phases of wound healing (Ashrafi et al., 2016). In order to prevent excessive scar formation, the majority of proliferative cells, including fibroblasts, inflammatory cells, and vascular cells, decrease in number in the area of the wound owing to apoptosis (Gonzalez et al., 2016). Type I collagen eventually replaces type III collagen as the ECM matures, increasing scar strength (Gurtner et al., 2008).

## 3 Types of neuropeptides

### 3.1 Substance P (SP)

Substance P (SP) is one of the main neuropeptides that C-nociceptive fibers release in response to injury. SP is an 11-amino acid neuropeptide encoded by the *TAC1* gene (Zieglgänsberger, 2019). It is produced in the dorsal root ganglion, whereafter it is disseminated centrally to the dorsal horn of the spinal cord and peripherally to the nerve ends of sensory neurons in the dermis and epidermis. It is present throughout the central and peripheral neurological systems, including the skin (Mashaghi et al., 2016). NK-1R, a neurokinin G-protein coupled receptor that is expressed in peripheral tissues and neurons, controls the activities of SP (Ständer and Yosipovitch, 2019). The SP synthesized by the peripheral nerves migrates upward to the posterior horn of the spinal cord and is retrogradely transported downward to the sensory nerve endings of the skin, muscles, bones, joints, blood vessels, and internal organs. It is mainly distributed in the Aδ nociceptive nerves and unmyelinated C fibers (Marek-Jozefowicz et al., 2023). Besides being mainly distributed in the nervous system, SP receptors can also be expressed by mast cells, macrophages, and fibroblasts. The SP endotoxin lipopolysaccharide (LPS) released from the nerve endings can induce the generation and release of SP by the above-mentioned cells (Lim et al., 2017). The biological activity of SP is mediated by the G protein-coupled receptor - NK1 receptor. Through the activation of the cell membrane phospholipase C (PLC) to generate 1,4,5-triphosphate inositol (IP3) and diacylglycerol (DAG), the intracellular calcium concentration is increased, thereby activating the serine/threonine-sensitive protein kinase C (PKC) (Falkenburger et al., 2013; Harris et al., 2022; Zhang et al., 2023). Out of all the neuropeptides linked with wound healing, SP has proven to be a powerful modulator of cutaneous wound healing. Both *in vitro* and *in vivo* tests have shown that SP has pro-angiogenic activity, and, more critically, SP has been found to play a crucial role in the infiltration of polymorphonuclear leukocytes into wound sites (Leal et al., 2015). By raising the levels of BCL-2 and proliferating cell nuclear antigen in burn wounds, SP also encourages fibroblast proliferation and inhibits apoptosis (Jung et al., 2016).

SP can affect different stages of wound healing. During the inflammatory stage, SP can activate immune cells, such as mast cells and macrophages, and facilitate the release of inflammatory mediators, like histamine, cytokines, and chemokines. Simultaneously, SP exerts its effect in the inflammatory response through the neurokinin 1 receptor (NK1R), influencing the recruitment and activation of immune cells (Suvas, 2017). In the proliferation stage, SP can stimulate the proliferation and migration of fibroblasts and keratinocytes, which is crucial for promoting wound repair and regeneration. Concurrently, by enhancing the synthesis of the extracellular matrix, such as collagen, it plays a role in the strength and structural integrity of the wound (Redkiewicz, 2022). In the wound remodeling stage, SP participates in the remodeling process of the extracellular matrix by influencing the expression and activity of matrix metalloproteinases (MMPs). SP may also affect wound maturation and scar formation by regulating growth factors, such as the transforming growth factor-β (TGF-β) family (Krizanova et al., 2022).

### 3.2 Calcitonin gene-related peptide (CGRP)

Calcitonin gene-related peptide (CGRP), a 37-amino acid peptide, has a significant perivascular location and is broadly expressed in neural tissue, including peripheral sensory nerves (Russell et al., 2014). CGRP exerts its effects via the calcitonin gene related peptide receptor (CGRPR), which is expressed in epidermal cells such as keratinocytes and melanocytes (Hou et al., 2011), which, upon forming a complex with the receptor activity-modifying protein (RAMP1), exerts its biological function by activating adenylate cyclase and elevating the intracellular cAMP concentration (Lu et al., 2024). These receptors are predominantly expressed in neutrophils, monocytes, and macrophages. CGRP activates Gq/11 protein and thereby influences multiple intracellular signaling pathways, such as the activation of PKC (protein kinase C), which contributes to the regulation of cell functions (Umoh et al., 2014). Furthermore, CGRP can also accelerate wound healing by promoting the release of TSP-1 (thrombospondin-1) to inhibit the recruitment of immune cells and expedite their death (Lu et al., 2024). Regarding upstream regulatory factors, inflammatory factors such as LPS (lipopolysaccharide) can stimulate DRG (dorsal root ganglion) neurons to release CGRP, suggesting that inflammatory responses may regulate the release of CGRP by influencing neuronal activities (de Souza et al., 2024). Downstream regulatory factors include the NLRP3 inflammasome and its downstream inflammatory factors, which play a crucial role in modulating the inflammatory responses elicited by CGRP (Zhu et al., 2023). According to recent research, the influence of CGRP on angiogenesis modulates its relationship with wound healing (Younan et al., 2010). Furthermore, co-application of CGRP and SP to human skin has been shown to result in dose-dependent long-lasting vasodilation, indicating a synergistic impact of the two neuropeptides (Schlereth et al., 2016).

In the inflammatory stage, CGRP facilitates wound healing by modulating the functions of immune cells. Researches have demonstrated that CGRP can act on neutrophils and macrophages via its receptor complex RAMP1, suppressing the recruitment of these cells and accelerating their demise, concurrently enhancing phagocytosis and polarizing macrophages into a pro-repair phenotype (Lu et al., 2024). Furthermore, CGRP can also regulate local inflammatory responses by inhibiting the release of certain pro-inflammatory chemokines (Yang et al., 2022). During the proliferation stage, CGRP exerts a promotional effect on the proliferation and migration of fibroblasts. *In vitro* experiments reveal that CGRP can facilitate the proliferation and migration of fibroblasts, augment the expression of collagen I, and alter the ratio of collagen I to collagen III (Chéret et al., 2014). Moreover, CGRP is also capable of accelerating the proliferation of keratinocytes and enhancing the speed of local wound contraction (Shi et al., 2013). In the remodeling stage, CGRP expedites wound healing by facilitating neovascularization. Studies have revealed that CGRP is capable of promoting angiogenesis, which is conducive to the formation of new tissues required during the wound healing process (Yang et al., 2022). Furthermore, CGRP is also implicated in regulating immune cell functions, especially under conditions of dysregulated neuroimmune interactions such as diabetes, by promoting the release of TSP-1 to inhibit the recruitment of immune cells and enhance their death, thereby accelerating wound healing (Lu et al., 2024).

### 3.3 Neuropeptide Y (NPY)

One of the most prevalent neurotransmitters in the central and peripheral nervous systems of mammals, NPY is a highly conserved 36 amino-acid polypeptide involved in wound repair (Jakobsson et al., 2019). The receptors of NPY belong to the G protein-coupled receptor (GPCR) family. In the human body, there are mainly five subtypes of NPY receptors: Y1R, Y2R, Y3R, Y4R, and Y5R (Yang et al., 2018). NPY receptors are expressed in multiple cell types, including immune cells, vascular endothelial cells, and neurons, among others. For instance, Y1R is expressed at higher levels in macrophages and T cells, but is scarcely expressed in B cells (Yu et al., 2022). Y2R is extensively expressed in newly formed blood vessels, and in mouse models lacking Y2R, a decrease in new blood vessels was observed, resulting in delayed skin wound healing (Ekstrand et al., 2003). Additionally, vascular endothelial cells also express Y1R, and they themselves express NPY, forming a positive feedback mechanism (Matsuda et al., 2002). NPY, by binding to its receptors, induces an increase in intracellular calcium concentration and the activation of MAPK1/MAPK3, while actively participating in AKT signaling, which is of paramount importance for regulating cell migration, particularly in wound healing. NPY also interacts with G proteins (such as Gαi1) via Y1R, thereby influencing downstream signaling pathways (Park et al., 2022). In addition to the nerves, the liver, spleen, megakaryocytes, and endothelial cells (ECs) have all been found to produce NPY (Shende and Desai, 2020). NPY participates in both the angiogenic and inflammatory phases of wound healing. An important delay in cutaneous wound healing and reduced neovascularization has been observed in genetically altered mice with deletion of the NPY-2R-encoding gene (Ekstrand et al., 2003).

In the inflammation stage, NPY promotes the generation of tumor necrosis factor alpha (TNF-α) elicited by LPS in macrophages via Y1R (Sun et al., 2020). Additionally, NPY is capable of regulating the secretion of inflammatory factors by macrophages, encompassing the inhibition of interleukin-1β (IL-1β) release from peritoneal macrophages (Zhou et al., 2013). These effects contribute to modulating the inflammatory response and preventing tissue damage caused by excessive inflammation. In the proliferation stage, NPY facilitates wound healing by exerting influences on immune cells and angiogenesis. Research indicates that NPY can regulate the secretion of TNF-α by macrophages through β-arrestin 2, thereby impacting the inflammatory response during the wound healing process (Singer et al., 2013). Simultaneously, NPY is also involved in the angiogenesis process, promoting the formation of new blood vessels to supply the necessary nutrients and oxygen to the wound (Zhang et al., 2021). At this stage, the proliferation and migration of fibroblasts are also affected by NPY, and these cells rebuild the damaged tissue by synthesizing collagen and other matrix proteins (Thangaratnarajah et al., 2017). In the remodeling stage, NPY continues to function by regulating the synthesis and degradation of collagen to enhance the quality of scar tissue. NPY promotes the cross-linking and alignment of collagen fibers and strengthens the tensile strength of the newly formed scar tissue (Fu et al., 2020). Furthermore, NPY might further promote wound contraction and tissue remodeling by influencing the expression of cytoskeletal α-actinin (Thiruvoth et al., 2015).

### 3.4 Vasoactive intestinal peptide (VIP)

VIP belongs to a class of structurally related peptides that also includes the hormones glucagon, secretin, and growth hormone-releasing hormone. These peptides all function by binding to the same family of receptors, which are found throughout the central and peripheral nervous systems (Harmar et al., 2012). The receptors of VIP are primarily VPAC1 and VPAC2, both of which pertain to the G protein-coupled receptor (GPCR) category (Tanaka et al., 2023). VIP receptors are expressed in multiple cell types, encompassing intestinal epithelial cells, neurons, and skin cells, among others (Ständer et al., 2004). VIP activates downstream signaling pathways via its receptors, predominantly involving the cAMP-PKA (protein kinase A) pathway and the AKT pathway. Upon binding to the VPAC1 receptor, VIP activates the Gs type of G protein, subsequently facilitating the generation of cAMP. cAMP then activates PKA, ultimately influencing the expression of downstream target proteins (Tasma et al., 2024). VIP can also activate the AKT signaling pathway through the VPAC2 receptor, thereby suppressing the inflammatory response and promoting cell proliferation (Castorina et al., 2014). VIP immunoreactivity is observed in the nerve fibers of the skin that lie close to capillaries, sweat glands, apocrine glands, meibomian glands, and hair follicles (Chéret et al., 2013). Adenosine 3′,5′-cyclic monophosphate (cAMP) is required for keratinocyte migration and proliferation during cutaneous wound healing, which is involved in re-epithelialization (Nuccitelli, 2003). It has been demonstrated that the paracrine activity of VIP and the autocrine activity of PACAP lead to keratinocyte proliferation (Granoth et al., 2000). Furthermore, VIP influences collagen remodeling during skin wound healing by modulating matrix metalloproteinase activity as well as promoting the production of NGF and inflammatory cytokines, such as IL-1 and IL-8, in keratinocytes (Chéret et al., 2014).

VIP possesses a remarkable anti-inflammatory effect. VIP acts on immune cells via specific membrane surface receptors, inhibiting the secretion of inflammatory factors such as nitric oxide (NO), interleukins (IL), and tumor necrosis factor (TNF)-α, and promoting the generation of the anti-inflammatory factor IL-10, thereby suppressing the excessive activation of T cells and maintaining the stability of the body’s immune status (Villanueva-Romero et al., 2018). VIP also plays a crucial role in the proliferative stage of promoting skin wound healing. VIP is capable of regulating the proliferation and differentiation of normal and tumor cells through its receptors. VIP receptors (VIPR) are widely present in various normal and tumor tissues. VIP binds to these receptors via VIPR, promoting cell proliferation and differentiation, thereby accelerating cell regeneration and repair during the wound healing process (Pankhaniya et al., 1998). VIP also exerts an important role in the remodeling stage of wound healing. VIP regulates the bidirectional communication between the immune and endocrine systems to maintain the body’s homeostasis (Martínez et al., 2019). VIP not only participates in the innate immune response but also engages in the adaptive immune response, promoting tissue remodeling during the wound healing process by regulating the functions of immune cells (Martínez et al., 2019).

## 4 Mechanisms underlying the role of neuropeptides in wound healing

### 4.1 Vascular dilation and vascular permeability

**FIGURE 1:**
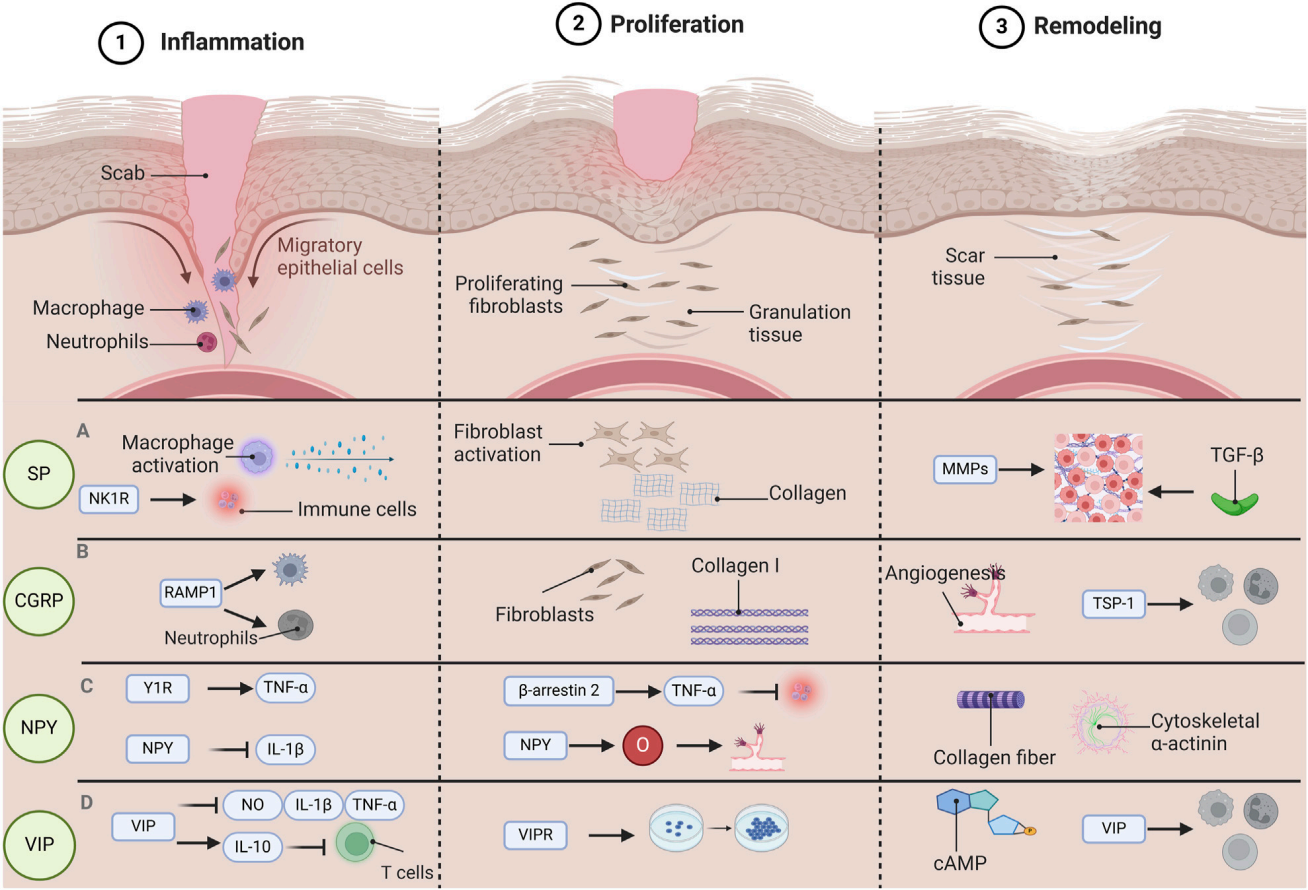
The function of various neuropeptides involved in the process of wound healing. Wound healing is categorized into three distinct phases: Inflammation, Proliferation, and Remodeling. Each phase is governed by unique pathological mechanisms, and various neuropeptides have specific functions throughout each stage of the wound healing. During the inflammatory phase, substance P (SP) has the capacity to activate macrophages and facilitate cytokine release. Concurrently, SP influences the recruitment and activation of immune cells via the neurokinin 1 receptor (NK1R). In the proliferative phase, SP promotes the proliferation and migration of fibroblasts, enhances the synthesis of the extracellular matrix (specifically collagen), and contributes to wound strength and structural integrity. During the remodeling phase, SP impacts the expression and activity of matrix metalloproteinases (MMPs) as well as transforming growth factor β (TGF-β), playing a role in the remodeling of the extracellular matrix. During the inflammatory phase, calcitonin gene-related peptide (CGRP) interacts with neutrophils and macrophages via its receptor activity-modifying protein (RAMP1), which inhibits their recruitment and accelerates their apoptosis. In the proliferative phase, CGRP facilitates the proliferation and migration of fibroblasts while enhancing the expression of collagen I. In the remodeling phase, CGRP promotes angiogenesis and expedites wound healing by inducing the release of thrombospondin-1 (TSP-1), thereby inhibiting immune cell recruitment and enhancing their apoptosis. In the inflammatory phase, neuropeptide Y(NPY) promotes the production of tumor necrosis factor α (TNF-α) through Y1R, and NPY is able to inhibit the release of interleukin-1β (IL-1β), which helps to modulate the inflammatory response and prevent tissue damage caused by excessive inflammation. In the proliferative stage, NPY can regulate TNF-α through β-arrestin 2, thereby affecting the inflammatory response during wound healing. At the same time, NPY also provides the necessary nutrients and oxygen to the wound, participates in the angiogenesis process, and promotes the formation of new blood vessels. During the remodeling phase, NPY promotes the cross-linking and alignment of collagen fibers and further promotes wound contraction and tissue remodeling by influencing the expression of cytoskeletal α-actin. During the inflammatory phase, vasoactive intestinal peptide (VIP) has the capability to suppress the release of inflammatory mediators such as nitric oxide (NO), interleukin (IL), and TNF-α, while simultaneously enhancing the synthesis of the anti-inflammatory cytokine IL-10. This action serves to prevent T cell overactivation and preserve the equilibrium of the immune system. In the proliferative phase, VIP facilitates cell growth and differentiation via vasoactive intestinal peptide receptors (VIPRs), thereby expediting cell regeneration and tissue repair during the wound healing process. In the remodeling phase, VIP contributes to tissue remodeling by influencing the activity of adenosine 3′,5′-cyclophosphate (cAMP) and modulating the functions of immune cells throughout the healing process. Abbreviations: SP, substance P; CGRP, calcitonin gene-related peptide; NPY, neuropeptide Y; VIP, vasoactive intestinal peptide.

Hemostasis, chemotaxis, and enhanced vascular permeability are characteristics of the inflammatory phase of wound healing, which serves to prevent further injury, close the wound, remove cellular debris and microorganisms, and promote cellular migration (Wang et al., 2018) (Figure 1). Vasomodulatory neuropeptides, such as SP and CGRP, are released when nerve endings are stimulated by trauma and the axonal reflex. These neuropeptides operate on retrocapillary venules to trigger inflammatory responses including vasodilation and increased permeability (Dimitriadou et al., 1997). Vascular dilatation causes an increase in blood flow to the site, resulting in erythema and heat, which are typical inflammatory symptoms. The transport of circulating cells and mediators to the site of injury is also accelerated by increased flow. Vascular permeability increases as a result of gaps developing between ECs as arteries dilate (Nagy et al., 2008). Vasodilation and enhanced permeability enable the movement of intravascular fluid, protein, and cellular components into the extravascular region, triggering the wound-healing process. Nitric oxide is required for effective wound healing (Frank et al., 2002). NO has the ability to widen blood vessels, encourage the growth of fibroblasts, and accelerate the healing of wounds (Wang et al., 2018). Additionally, NO promotes the proliferation of keratinocytes, fibroblasts, and endothelial cells, as well as collagen deposition. It also plays a role in the control of the immunological response and possesses broad-spectrum antimicrobial activity (Zhou et al., 2017). Owing to its direct actions on ECs and increased nitric oxide release, SP promotes vasodilatation and microvascular permeability (Baraniuk et al., 1990). CGRP increases plasma extravasation and is a vasodilator (Ashrafi et al., 2016). SP and CGRP can upregulate iNOS gene and protein expression, activate macrophage inducible NO synthase activity, and enhance NO production (Jeon et al., 1999).

However, there are differences in the mechanisms of vascular dilation and vascular permeability during the promotion of healing facilitated by CGRP and SP. CGRP is a highly effective vasodilator, capable of inducing a prolonged and sustained increase in epidermal blood flow (Schlereth et al., 2016). It acts on neutrophils, monocytes, and macrophages via receptor activity-modifying protein 1 (RAMP1), inhibiting the recruitment of these cells, accelerating their demise, enhancing endocytosis, and polarizing macrophages to a pro-repair phenotype (Lu et al., 2024). This persistent vascular dilation contributes to the supply of nutrients and oxygen required during the wound healing process. In contrast, SP induces only transient vascular dilation but causes significant plasma extravasation (Schlereth et al., 2016). This implies that while SP promotes vascular dilation, it also causes more fluid and proteins to exude from the blood vessels into the surrounding tissues. This may exert an adverse influence on wound healing and constitute one of the limitations of SP. SP primarily accelerates wound healing by promoting the aggregation and differentiation of epidermal cells (Peng et al., 2016).

### 4.2 Regulation of immune cell function

Studies have revealed that mast cells, T cells, and macrophages all possess neuropeptide receptors (Machelska, 2011; Hanč et al., 2023). Neuropeptides influence the production, activation, migration, and release of immune cells as well as the release of growth factors to regulate wound healing (Mehta and Granstein, 2019; Tamari et al., 2024). Via phagocytosis and the production of many proteases and cytokines, macrophages play a role in the control of wound healing. Upon macrophage removal, wound healing is markedly slowed (Willenborg et al., 2022). SP can promote the aggregation, phagocytosis, and chemotaxis of macrophages (Nicoletti et al., 2012). CGRP functions as a negative regulator of innate immune responses and helps to prevent tissue damage in inflammatory disorders by acting on macrophages through elevation of IL-10, IL-10-independent stimulation of the inducible cAMP early repressor (ICER), and suppression of NF-B activity (Holzmann, 2013). T lymphocytes play a role in the control of wound healing and the development of fibrous tissue (Hu et al., 2022). NPY controls both the innate and adaptive immune systems by modulating the release of cytokines from macrophages and helper T cells, antigen presentation, as well as the activation of natural killer (NK) cells and antibody formation (Pradhan et al., 2009).

The basis for the migration of T cells to the inflammatory site is their adherence to the ECM (Alcaide, 2022). CGRP, NPY, and SP may encourage T cells to bind to fibronectin (Levite et al., 1998). Furthermore, there exist differences in the mechanisms of regulation in immune cell function during the promotion of healing facilitated by CGRP, SP and NPY. SP promotes wound healing in trauma by enhancing the phagocytosis of macrophages and neutrophils and inducing the release of cytokines such as IL-1 and TNFα from these cells (Suvas, 2017). This effect has been found to be particularly remarkable in the healing of chronic wounds in diabetic patients (Zhang et al., 2022). CGRP can be released through nerve terminals to stimulate the activation of immune cells such as neutrophils and macrophages, thereby accelerating tissue repair (Suvas, 2017). On the other hand, CGRP also exhibits anti-inflammatory properties, capable of suppressing the activity of natural killer cells and the accumulation of neutrophils (Yu et al., 2023). CGRP can also exert immunosuppressive effects by inhibiting the antigen-presenting capacity of dendritic cells and reducing the proliferative response of T cells (Yu et al., 2023). Exogenous application of NPY can significantly improve chronic wound healing in diabetic mice (Stangerup et al., 2024). NPY regulates the functions of immune cells by activating specific receptors and promotes cell proliferation and differentiation during the wound healing process (Stangerup et al., 2024).

### 4.3 Cell proliferation and migration

After migrating from the skin appendage and wound edge, keratinocytes multiply, mature, and restore the epithelium’s barrier function (Kiya and Kubo, 2019). Fibroblasts play a crucial role in the development of granulation tissue, leading to the development of ECM, which in turn provides a structural framework for the production of new capillaries by ECs in a process known as angiogenesis. ECs are recruited to the wound area from the border of the wound or from the bone marrow (Profyris et al., 2012). By promoting DNA synthesis, SP has strong proliferative effects on fibroblasts, keratinocytes, and ECs (Kähler et al., 1996). SP aids in the remodeling of granulation tissue by encouraging dermal fibroblast migration and proliferation. It also stimulates the production of epidermal growth factor and its related receptor (Lai et al., 2002). CGRP encourages keratinocyte growth and migration, which leads to re-epithelialization, fibroblast growth, adhesion, and differentiation into myofibroblasts, all of which encourage granulation tissue remodeling (Chéret et al., 2014). VIP has been demonstrated to be a regulator of keratinocyte migration and a growth factor for keratinocyte proliferation. Recently, it was discovered that in samples of difficult-to-heal wounds treated with platelet lysate, NPY produced from platelet lysate affected the migratory and angiogenic capability of human adipose-derived stromal cells and co-localized with endothelial markers CD31 and VEGF (Businaro et al., 2018). Although NPY is mostly recognized to be involved in the healing of tendons and cartilage, it also affects cutaneous healing via the pro-angiogenic receptors NPY-2R and NPY-5R (Salo et al., 2008). To sum up, SP, CGRP, NPY, and VIP mainly exert significant roles in skin wound healing by facilitating the proliferation and migration of different types of cells.

### 4.4 Angiogenesis

To address the metabolic needs of the highly proliferative healing tissue, new blood vessels are formed during the angiogenesis process in the proliferation stage. Microvascular endothelial cells multiply and move into the wound bed in response to these modifications, sprouting new vessels that join with existing ones to form sturdy tubular networks (Veith et al., 2019). Nitric oxide may be a mediator by which SP induces angiogenesis (Muangman et al., 2009). *In vitro* and *in vivo* studies demonstrate that CGRP has a potent dilating effect on blood arteries and influences angiogenesis and EC proliferation (Mapp et al., 2012). When NYP increases, angiogenic factor, an NPY downstream target, directly encourages EC migration and proliferation as well as neovascularization (Dumont et al., 2015). In addition, NPY can be produced from platelet lysates to promote angiogenesis and EC proliferation in a calcium-dependent manner (Businaro et al., 2018). VIP promotes VEGF synthesis through the extracellular signal-regulated kinase (ERK) 1/2 and p38 mitogen activated protein kinase (MAPK) signaling pathway, especially in skin keratinocytes, to exert angiogenic effects both *in vitro* and *in vivo* (Yu et al., 2010).

SP, CGRP, NPY and VIP display certain disparities in the molecular mechanisms of promoting angiogenesis during the healing process of various types of wounds. Both SP and CGRP facilitate wound healing by enhancing neovascularization and suppressing certain pro-inflammatory chemokines (Shi et al., 2024). CGRP is predominantly released by sensory neurons and interacts with immune cells to furnish signals for wound repair (Lu et al., 2024). It is persistently secreted after skin and soft tissue injuries, promotes angiogenesis and nourishes the skin, thereby facilitating wound re-epithelialization and scar healing (Wu et al., 2018). Moreover, CGRP is also involved in vascular regulation and angiogenesis by influencing mast cells, endothelial cells, fibroblasts and keratinocytes (Zhu et al., 2024). NPY promotes angiogenesis through interacting with immune cells. The NPY/Y2/DPPIV pathway also assumes a significant role in vascular regeneration among the elderly, yet its angiogenic capacity diminishes with advancing age (Zhang et al., 2021).

## 5 Limitations of neuropeptides and corresponding strategies

The limitations of SP are typically regarded as having pro-inflammatory actions, as it participates in the initial vascular inflammatory response after UV irradiation of the skin, involving vasodilation and plasma extravasation, which leads to edema and erythema. Furthermore, although exogenous application of SP can accelerate wound closure and promote cell migration, its long-term usage might impose a burden on the skin tissue and cause fibrosis or other structural alterations (Um et al., 2016). Therefore, while employing SP to facilitate wound healing, the concurrent use of anti-inflammatory agents may be beneficial in modulating inflammatory responses and mitigating tissue damage (Freedman et al., 2023). For chronic wounds, integrating additional therapies such as calcium-based nanoparticles could further enhance the efficacy of wound healing and prevent hypertrophic scarring (Mei and Wu, 2022).

Owing to the role of CGRP in promoting angiogenesis and the behavior of immune cells, if it is misused, there might be an augmented risk of chronic wounds, particularly among diabetic patients (Shi et al., 2024). Furthermore, although CGRP can suppress the release of certain pro-inflammatory chemokines, excessive or inappropriate utilization may elicit an improper inflammatory response, thereby influencing the wound healing (Shi et al., 2024). Hence, its effect can be regulated by employing CGRP antagonists or engineered versions of CGRP, which can diminish its potential negative impacts while not completely inhibit its beneficial effects (Toda et al., 2008). Simultaneously, individualized therapy should be administered in accordance with the specific circumstances of the patient (such as diabetic status, neuropathy, etc.,) to guarantee that the application of CGRP can facilitate healing without causing adverse reactions.

The pro-angiogenic function of NPY, although exerting a positive impact on vascular recanalization and wound healing in ischemic tissues, may concurrently induce negative influences such as tumorigenesis and vision disorders. Additionally, the weakened capacity of angiogenesis in the elderly results in a diminished NPY-mediated angiogenic ability, further restricting its efficacy in wound healing (Reichmann and Holzer, 2016). Consequently, in clinical applications, through local administration of NPY and strict control of its dosage to avert excessive utilization, the extensive effects on the systemic level can be mitigated, thereby lowering the risks of tumors and vision issues (Wang et al., 2020).

Prolonged utilization of VIP might result in the emergence of drug resistance, particularly in the management of chronic wounds. If VIP is combined with other antibiotics or anti-inflammatory drugs, the risk of drug-resistant bacterial strains may escalate (Werdin et al., 2009). Through the collaboration of a multidisciplinary team and comprehensive consideration of the patient’s nutritional status, infection control, and overall health condition, individualized treatment regimens can be formulated. For instance, integrating adjunctive therapeutic approaches such as nutritional supplementation, negative pressure wound therapy, and traditional Chinese medicine treatments can enhance the therapeutic efficacy of VIP and mitigate its side effects (Liu et al., 2023).

## 6 Neuropeptide-derived treatments in cutaneous wound healing

Numerous therapeutic strategies, such as the use of therapeutic dressings (Boateng and Catanzano, 2015), laser therapy (Farivar et al., 2014), and hyperbaric oxygen (Kranke et al., 2015), have been developed to promote wound healing. Among these, at present, as demonstrated in Table 1, extensive focus has been directed to neuropeptide-derived therapies, particularly those based on SP.

**TABLE 1:** Summary of studies indicating neuropeptides derived treatments.

Treatment type	Ingredients/parameters	Mode of action	Features	Advantages	Results	References
Hydrogel	Antioxidative sodium thiosulfate, surfactant polysorbate 80, hydroxyethyl cellulose (HEC) gelling agent	Cell proliferation and migration	SP at doses of 1–10 μg/mL in hydrogel was stable at 4°C, 37°C and 60°C for up to 4 weeks	Area of re-epithelization was larger and the density of granulation tissue, fibroblast and collagen was higher	SP gel increased the proliferation and migration of human epidermal keratinocyte (HEK) and of human dermal fibroblasts (HDF)	Kim et al. (2018), Kim et al. (2019)
Hydrogel	Laponite and sodium polyacrylate	Cell proliferation and migration	Nonswellable, self-standing, biodegradable and biocompatible, and the simple fabrication process with mild conditions have enabled the encapsulation of controlled concentrations of SP	One-time application of it at 10 µM induced 98% wound closure within 16 days	SP was successfully released from the hydrogel and stimulated the keratinocytes to proliferate and migrate, leading to re-epithelialization	De Serres-Bérard et al. (2020)
Hydrogel	Chitosan hydrochloride-coated liposomes	Cell proliferation and migration	Delayed and slower release of SP	Better efficacy than free SP	The SP-CH-LP was not toxic for HaCaT keratinocytes and stimulated their proliferation in a concentration-dependent manner	Mengoni et al. (2017)
Hydrogel	Chitosan hydrochloride and mixed with HEC	Angiogenesis	A stable delivery system for SP	An enhanced capacity to repair full-thickness skin defects	Strengthened the vascularization, extracellular matrix deposition and remodeling, and nerve regeneration promoting efficient recovery of the skin defects in acute wounds	Li et al. (2021)
New Dressing	Coated SP-loaded ZIF-8 nanoparticles (SP@ZIF-8) with polyethylene glycol-thioketal (PEG-TK) to fabricate SP@ZIF-8-PEG-TK nanoparticles, and encapsulated them in injectable hydrogel composed of sodium alginate and pectin and cross-linked using calcium chloride	Regulation of immune cell function	A novel SP-delivery system using zeolite imidazolate framework-8 (ZIF-8) nanoparticles	High SP-loading efficiency	Promote an early inflammatory response and subsequent M2 macrophage polarization in the wound-healing process	Zhu et al. (2020)
Electrical stimulation (ES)	Degenerate waves ES waveform at 100 mV/mm (60 Hz)	Cell proliferation and migration	Non-invasive and pain-free	Reduce medical costs	Increase in substance P expression and number of substance P þ cells in wounded human skin after ES	Sebastian et al. (2017)
ES	Anodal and cathodal HVMPC, respectively (154 μs 100 Hz; 360 μC/second; 1.08 C/day), 50 min per day, 5 days per week, for a maximum of 8 weeks	Angiogenesis	Non-invasive and pain-free	Reduce medical costs	ES improved blood flow and wound area reduction rate	Ashrafi et al. (2017)
Skin-derived precursors (SKPs)	Skin-derived precursor cells isolated from diabetic murine skin were cultured in sphere formation medium	Angiogenesis	Multipotent adult stem cells with the tendency to differentiate into neurons	Increased efficiency of angiogenesis	Skin-derived precursor cells promoted diabetic wound healings through vasculogenesis at the early stage of wound healing	Sato et al. (2015)

### 6.1 Hydrogels

Hydrogels, which are three-dimensional hydrophilic polymeric networks, have the capacity to absorb huge volumes of water or biological fluids. They are created through crosslinking and polymerization. Their biocompatibility, biodegradability, and ability to entrap huge numbers of molecules make hydrogels a popular choice for the delivery of several medications (Redkiewicz, 2022). Various proteases preferentially degrade SP, and its half-life is relatively brief, ranging from seconds to minutes (Mashaghi et al., 2016). To increase the stability of SP, hydrogels are frequently produced as SP formulations. A SP-based hydrogel was created by Kim et al. (Kim et al., 2018; Kim et al., 2019) by combining the antioxidant sodium thiosulfate with the surfactant polysorbate 80 and a gelling ingredient called hydroxyethyl cellulose (HEC). *In vitro*, SP gel promoted human epidermal keratinocyte (HEK) and human dermal fibroblast (HDF) proliferation and migration, which improved wound healing. Using an SP-based hydrogel with a Laponite nanodisc, De Serres-Bérard et al. (2020) was able to induce 98% wound closure in just 16 days after a single application. This suggests that the SP-based hydrogel stimulated keratinocyte proliferation and migration, which in turn led to re-epithelialization. Hydrogels offer a variety of distribution options that can improve wound healing, as detailed in Table 1 (Mengoni et al., 2017; Li et al., 2021).

### 6.2 New dressing

To enhance the stability and distribution of SP in wound healing, Zhu et al. (2020) created a novel dressing comprising SP@ZIF-8-PEG-TK@CA nanoparticles. Results revealed that SP@ZIF-8-PEG-TK@CA nanoparticles increased the expression levels of inflammation-related genes in macrophages, stimulated the growth of human dermal fibroblasts (HDF), displayed favorable cytocompatibility, and demonstrated excellent wound-healing efficacy by encouraging an early inflammatory response and subsequent M2 macrophage polarization in the wound-healing process. New dressings derived from neuropeptides remain to be further developed and studied.

### 6.3 Electrostimulation (ES)

There is increased interest in electrotherapy, often known as ES, as a painless, non-invasive procedure that is known to encourage skin healing. According to extant studies (Kai et al., 2017; Sebastian et al., 2015), ES can also increase keratinocyte proliferation and dermal angiogenesis, collectively accelerating the healing process. The idea behind ES is to release the appropriate neuropeptides while simulating the endogenous electric current that occurs during wound healing (Ashrafi et al., 2017). Sebastian et al. (Sebastian et al., 2017) demonstrated that ES promotes wound healing by increasing the expression of SP neuropeptide and indicators of neuronal development, including PGP9.5 and TUBB3, within the wound. Both *in vitro* and *in vivo* studies have revealed that ES enhances the regeneration of peripheral sensory axons (Wood and Willits, 2009). To assess the effects of high voltage pulse current on wound skin blood flow and stress ulcer healing in patients with nerve injuries, Polak et al. (2018) performed a randomized, controlled, double-blind clinical study and found that electrical stimulation significantly increased the rate of blood flow to the wound and accelerated the healing of the affected area.

### 6.4 Skin-derived precursors (SKPs)

Using SKPs or induced pluripotent stem cells (iPS) developed into neurons, a potential supply of human neuropeptides, ideally from sensory neurons, could be created (Denham et al., 2015). In a model of nerve injury, SKPs themselves or SKP-derived Schwann cells are capable of causing remyelination and regeneration of peripheral nerves, which increases the release of neuropeptides (Grochmal et al., 2014). According to research by Sato et al. (2015), intradermal injections of SKPs around full-thickness excisional cutaneous wounds in diabetic mice facilitated more rapid wound closure and re-epithelization as well as earlier angiogenesis, and may, therefore, support wound reinnervation. Similarly, transplanting SKPs into a collagen sponge improved wound healing in diabetic rats, with this benefit likely being attributable to improved angiogenesis (Ke et al., 2015).

### 6.5 Neuropeptide drugs

Research indicates that SP can be applied topically either alone or in combination with curcumin, significantly accelerating wound closure and reducing the mRNA expression of TNF-α, IL-1β, and MMP-9, while significantly increasing the expression of IL-10 (Redkiewicz, 2022). The combination of SP with dimethyloxalylglycine (DMOG) when used in a hydrogel can markedly promote cell proliferation and significantly enhance *in vivo* wound healing (Wang et al., 2023). Besides, SP ointment (trade name: Nerve Tonic Ointment) functions by increasing its stability in lipid-based systems, enabling SP to gradually infiltrate the patient’s skin and muscles, thereby facilitating wound healing (Lu, 1985). Moreover, additional research suggests that Shengji Xiangpi Ointment may alleviate pain and promote wound healing by influencing the contents of NGF, SP, and S100A8/A9 in wounds (Lu et al., 2019). Engineered CGRP has been demonstrated to accelerate wound healing and facilitate muscle regeneration in mice lacking nociceptors and in diabetic mice suffering from peripheral neuropathy (Lu et al., 2024). VIP exhibits a certain role in mediating corneal wound healing, and exogenous VIP partially reverses the impairment to corneal wound healing induced by resin toxin (Zhang et al., 2020).

### 6.6 Gene therapies

The introduction of SP-related genes into the wound area through gene delivery systems enables the long-term regulation of SP expression, thereby facilitating wound healing (Oryan et al., 2019). Moreover, the research on novel nucleic acid delivery systems also offers technical support for SP gene therapy (Fan et al., 2022). Studies have demonstrated that direct injection of the CGRP gene can facilitate wound healing in rats. After injecting CGRP, by examining the influence of wound exudate on fibroblast growth at different days post-injury, the alteration patterns of various cytokines in the wound microenvironment in regulating the growth of repair cells were explored, which provides a reference for the clinical application of neuropeptides in treating local wounds (Sun, 1998).

## 7 Summary and outlook

Neuropeptides play a pivotal role in the process of wound healing, and the potential underlying mechanisms may be related to vascular dilation and vascular permeability, regulation of immune cell function, cell proliferation, migration, as well as angiogenesis, as detailed in Table 2. Furthermore, neuropeptides may have pathophysiological impacts on wound healing, although these effects are not yet recognized. The majority of treatments based on neuropeptides emphasize the use of topical applications such as novel dressings or delivery systems including hydrogels. Recent research has demonstrated the potential applications of SKPs and ES. Additional studies of neuropeptides may enhance understanding of the mechanisms underlying wound healing and open up new possibilities for treating pathological scars and accelerating skin repair. Neuropeptides exhibit distinct roles in various stages of wound healing. However, overall, they demonstrate the potential to facilitate healing. Personalized neuropeptide therapy, by targeting specific neuropeptides and receptors, can offer novel therapeutic strategies for chronic wounds and other non-healing tissues. Incorporating other therapeutic approaches, such as immune modulation and cell therapy, can further enhance the therapeutic efficacy and bring hope to patients.

**TABLE 2:** Classification of neuropeptides.

Neuropeptides	Structures	Wound healing stages	Primary functions	References
Substance P (SP)	Undecapeptide	Inflammation, proliferation, remodeling	In inflammation stage, SP can activate immune cells and facilitate the release of inflammatory mediators. In proliferation stage, it plays a role in the strength and structural integrity of the wound. In wound remodeling stage, SP participates in the remodeling process of the extracellular matrix	Redkiewicz (2022)
Calcitonin gene-related peptide (CGRP)	Composed of 37 amino acids	Inflammation, proliferation, remodeling	In inflammatory stage, CGRP can modulate the functions of immune cells. In proliferation stage, CGRP exerts a promotional effect on the proliferation and migration of fibroblasts. In remodeling stage, CGRP can promote angiogenesis	Chéret et al. (2014)
Neuropeptide Y (NPY)	Composed of 36 amino acids	Inflammation, proliferation, remodeling	In inflammation stage, NPY can promote the generation of TNF-α. In proliferation stage, NPY is involved in the process of angiogenesis. In remodeling stage, NPY can regulate the synthesis and degradation of collagen	Singer et al. (2013)
Vasoactive intestinal peptide (VIP)	Composed of 28 amino acids	Inflammation, proliferation, remodeling	In inflammation stage, VIP acts on immune cells to inhibit the secretion of inflammatory factors and promote the generation of the anti-inflammatory factor. In proliferative stage, VIP can regulate the proliferation and differentiation of normal and tumor cells. In remodeling stage, VIP can regulate the bidirectional communication between the immune and endocrine systems to maintain the body’s homeostasis	Martínez et al. (2019)

Although exogenous neuropeptides have been proved to be capable of promoting the healing of refractory wounds, how to select the appropriate neuropeptides and the doses still remains a challenge. The differences in axonal growth and function of different nerve fibers also exert an influence on the efficacy of neuropeptides. Additionally, the application of denervation measures such as neuropeptide antagonists or highly selective nerve transection in alleviating scar hyperplasia requires further investigation. Research on neuropeptide receptors is also a new direction for future studies. Although ion channels directly gated by neuropeptides have been identified, offering the possibility of developing a new generation of neuroscience tools, how to utilize these receptor-specific alterations in the membrane potential of neurons to regulate neuropeptide release and action more effectively still awaits further exploration.
